# Dr. B. H. Teague's Depressed Engine Disks

**Published:** 1886-04

**Authors:** 


DR. B. H. TEAGUES' DEPRESSED ENGINE DISKS.—Dr. Teague, of Aiken, S. C., has devised a new disk with a depressed centre, which answers the purpose of finishing fillings on approximal surfaces and near cervical portions of the tooth crowns in a very satisfactory manner. We are certain that all who use them will find that they are a great improvement over the flat disk.

